# A Cyber-Physical System for Girder Hoisting Monitoring Based on Smartphones

**DOI:** 10.3390/s16071048

**Published:** 2016-07-07

**Authors:** Ruicong Han, Xuefeng Zhao, Yan Yu, Quanhua Guan, Weitong Hu, Mingchu Li

**Affiliations:** 1 State Key Laboratory of Coastal and Offshore Engineering, Dalian University of Technology, Dalian 116000, China; hanruicong@mail.dlut.edu.cn; 2 School of Civil Engineering, Dalian University of Technology, Dalian 116000, China; 3 School of Electronic Science and Technique, Dalian University of Technology, Dalian 116000, China; yuyan@dlut.edu.cn; 4 School of Software Technology, Dalian University of Technology, Dalian 116000, China; xinmuheart@163.com (Q.G.); hu_weitong@foxmail.com (W.H.); mingchul@dlut.edu.cn (M.L.)

**Keywords:** cyber-physical systems, offshore hoisting monitoring, smartphone sensors, accelerometer, gyroscope

## Abstract

Offshore design and construction is much more difficult than land-based design and construction, particularly due to hoisting operations. Real-time monitoring of the orientation and movement of a hoisted structure is thus required for operators’ safety. In recent years, rapid development of the smart-phone commercial market has offered the possibility that everyone can carry a mini personal computer that is integrated with sensors, an operating system and communication system that can act as an effective aid for cyber-physical systems (CPS) research. In this paper, a CPS for hoisting monitoring using smartphones was proposed, including a phone collector, a controller and a server. This system uses smartphones equipped with internal sensors to obtain girder movement information, which will be uploaded to a server, then returned to controller users. An alarming system will be provided on the controller phone once the returned data exceeds a threshold. The proposed monitoring system is used to monitor the movement and orientation of a girder during hoisting on a cross-sea bridge in real time. The results show the convenience and feasibility of the proposed system.

## 1. Introduction

Cyber-physical systems (CPS) have recently become an important research field these years [1,2]. It’s a multidisciplinary field involving physics, communication, computation, control, and so on. CPS tries to connect the physical world and information world. It not only serves as the ears and eyes of the information with sensors, but also serves as the hand to change the mode of the physical activity and provide convenient lives for people [3,4]. There have been many CPS applications [5,6], such as medical devices and systems, assisted living, traffic control and safety, advanced automotive systems, process control, distributed sensing command and control, smart structures, autonomous electric vehicle [7] and so on.

In recent years, smartphones have become popular on an unprecedented scale and can now be used as effective scientific tools. With an operating system, communication system and built-in sensors, they have already been applied to cyber-physical systems [8], including human health monitoring [9], vehicle maintenance services [10] and accident detection [11], motivation recognition [12,13,14,15], and structural health monitoring (SHM) [16,17,18,19,20,21,22,23,24]. Authors have also applied smartphones in the SHM field [25], in uses such as cable force monitoring [26,27,28], displacement monitoring [29], and earthquake rescue [30,31], etc. These applications allow the people to participate in the structural health perception process through the mobile phone and help people take corresponding measures to be safer.

During the construction of cross-sea bridges, each operation faces challenges due to the complexity of the environment, particularly in terms of hoisting [32]. The girders that require hoisting into place are typically bulky and heavy, and the crane performing the hoisting operation is floating on the sea and is thus significantly affected by waves and winds. Therefore, offshore construction is considerably more difficult than construction on land. The hoisting of a heavy structural element is critical to the entire project construction plan; any accident during a hoisting operation could result in considerable property loss or casualties and affect the construction schedule of the entire project. To ensure that a hoisting operation can be completed safely, the orientation and movement of a girder should be monitored in real time, and corresponding measures should be taken based on monitoring results. For monitoring the girder hoisting process, the total station is employed in the traditional method. However, it’s always influenced by landform; some parts can’t be measured because of the block and it’s difficult to place it on the site construction at sea. Thus, a new sensing and monitoring method, which calls for fewer restrictions for landform and more convenient operations, is of great value. As previously mentioned, smartphones represent a mobile technology of the cyber-physical social system, with advantages of lower cost, convenience, easy operation, that may be useful for girder hoisting monitoring. We highlight the features and contributions of our paper as follows:

1. An iPhone-based monitoring system is developed; this system uses smartphones equipped with internal sensors to obtain girder movement information, which will be uploaded to a server, and then return to controller users.
2. The system consists of a controller and collectors. The controller can send instruments to the collector to control the state of collector, it can also receive monitoring and warning information from the collector in real-time.
3. An alarming function is designed, and once the returned data exceeds a threshold, an alarm will appear on the controller iPhone.
4. The proposed system is used to monitor the movement and orientation of a girder during hoisting on a cross-sea bridge. The site monitoring results validate the data acquisition, data transmission, commands control and alarming functions. This CPS using smartphones and wireless networks in hoisting monitoring can provide more field conditions for operators and help them take corresponding measures to ensure safety.

The remainder of the paper is organized as follows: Section 2 describes the offshore bridge which girder hoisting need to be monitored. Section 3 gives the architecture of the hoisting monitoring system, and calibrates the angle measured by the iPhone. Section 4 and Section 5 show the monitoring test on the side-span and middle-span respectively. Section 6 concludes this paper.

## 2. Engineering Description

**Figure 1:**
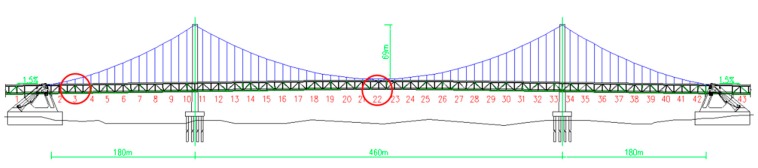
Elevation of the cross-sea bridge investigated in this study.

The primary bridge is a three-span, earth-anchored suspension bridge with a double tower. Its total span is 820 m with a primary span length of 460 m and side span lengths of 180 m each. The stiffening girder is a steel truss with a height of 10 m. There are 43 girders that must be hoisted. All girders are heavy and bulky steel truss structures with weights from 264.18 to 527.19 t. Girders in the side spans must be hoisted using a floating crane, and the girders in the middle span were lifted using two cranes stretched across the two primary cables. The hoisting procedures must be implemented in good weather and calm seas, and hoisting safety is an important factor that must be considered. The monitoring for hoisting is essential to make sure the safety, and can be realized in real-time and more convenient with the developed system. The elevation of the bridge is shown as Figure 1, and the girder numbers are labeled in red. In the following parts, the girders monitored were No. 3 in side-span and No. 22 in mid-span.

## 3. Hoisting Monitoring System

### 3.1. Monitoring System Design

The monitoring system consists of a collector program that is installed on an iPhone (i.e., the collector) and a controller program that is installed in another iPhone (i.e., the controller). Additionally, a web server is used to gather the data collected by the collector iPhones and then return data to the controller.

**Figure 2:**
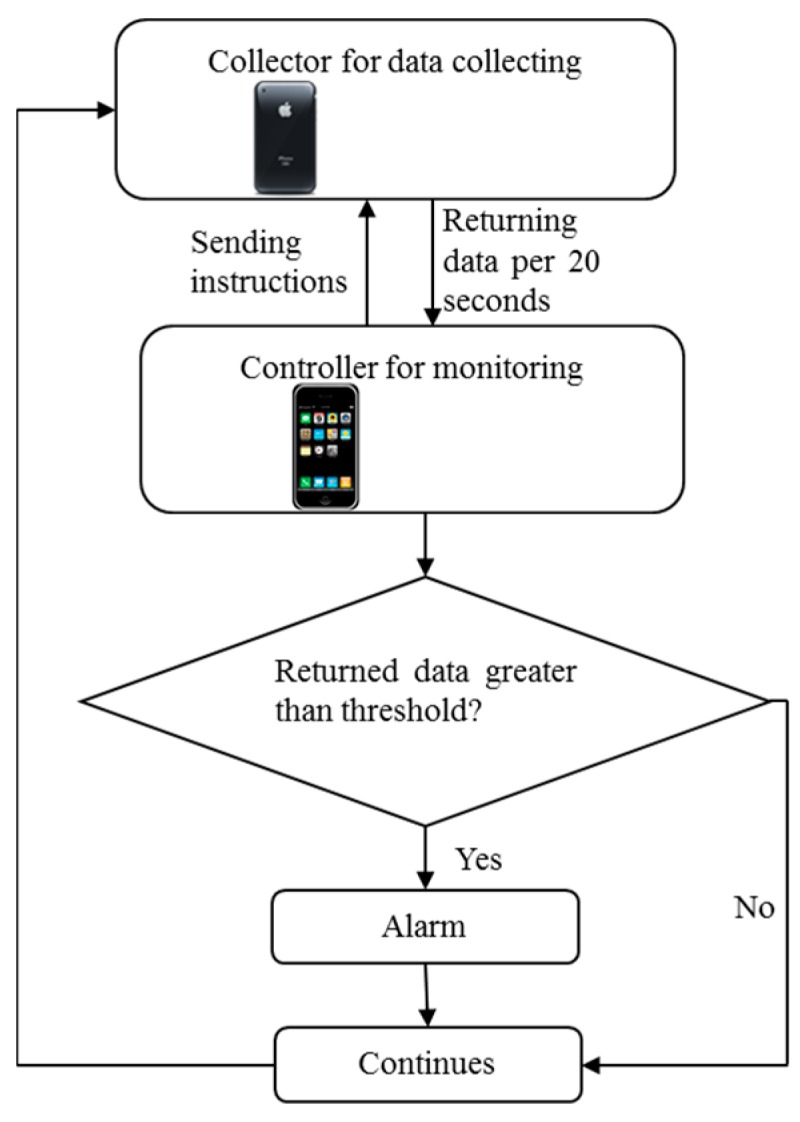
Flowchart of the monitoring and alarm systems.

The controller sends instructions to a collector and observes the collected data. The collector collects monitoring data through its built-in sensors after receiving instructions from the controller and returns the data to the controller every 20 s. Once the current returned data exceeds a given threshold (e.g., the threshold of acceleration is 10 m/s^2^, and the threshold of angle is 10°), an alarm will be triggered on the controller iPhone, which then continues collecting and monitoring. The threshold can be set on the controller at any time according to operator experience and the requirements of the on-site conditions. The flowchart of this system is presented in Figure 2.

### 3.2. Sensor Subsystem

#### 3.2.1. Sensor Parameters 

Three parameters are primarily considered during a hoisting process: the vertical acceleration and the angles of the *x*- and *y*-axes, which will be introduced in Section 3.5. The vertical acceleration is monitored to avoid the occurrence of a sudden drop; the angles noted are examined to monitor the locations of four corners of the structure during hoisting. Acceleration is collected by the built-in BMA220-type accelerometer (Bosch, Stuttgart, Germany), which allows measurement of acceleration in three perpendicular axes. This sensor is based on micro electro mechanical systems (MEMS). Additionally, the digital resolution is 6 bit. The acceleration sensor can be programmed to optimize functionality, performance and power consumption in customer-specific applications. The angle is determined by invoking the convert function in the iPhone to read the angular rate of rotation collected by the built-in L3G4200D-type gyroscope (ST, Geneva, Switzerland), which is also a MEMS-based sensor. The gyroscope integrates low- and high-pass filters with a user-selectable bandwidth and incorporates power-down and sleep modes, a temperature sensor, and first-in first-out memory. The basic features of the accelerometer and gyroscope in the iPhone are provided in Table 1. The mechanical characteristics of the accelerometer and gyroscope in the iPhone 5S are presented in Table 2 and Table 3, respectively.

**Figure 3:**
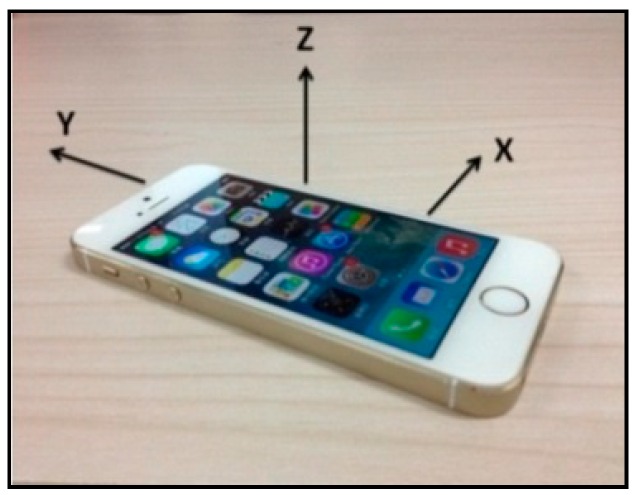
Acceleration directions.

**Figure 4:**
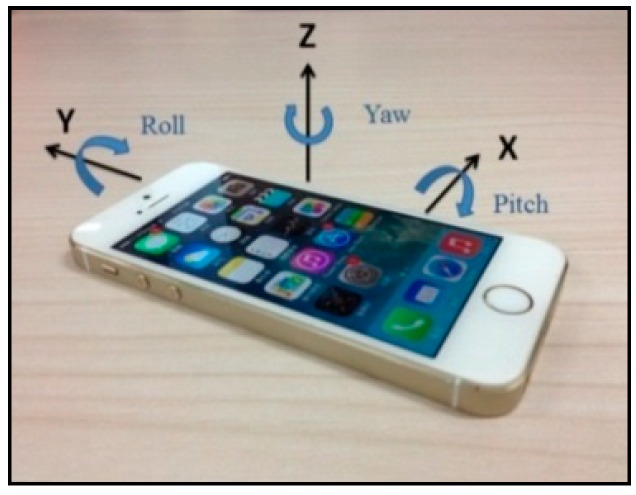
Angle-of-rotation directions.

Based on the preceding characteristics, the basic performances of the accelerometer and gyroscope are stable, and these devices can thus satisfy engineering requirements in certain situations. The acceleration direction is specified as follows: regardless of the position of the phone, when the screen faces the user, the horizontal direction (i.e., left to right) is the positive direction of the *x*-axis, while the vertical direction (i.e., bottom to top) is the positive direction of the *y*-axis. The direction that faces the user is the positive direction of the *z*-axis, which is perpendicular to the phone’s screen. The acceleration directions are shown in Figure 3. To determine the angle, the data obtained by the gyroscope is categorized into three groups (i.e., pitch, roll, and yaw), which represent the rotation angles around the *x*-, *y*-, and *z*-axes, respectively. The angle-of-rotation directions are shown in Figure 4.

#### 3.2.2. Calibration of Angle

**Figure 5:**
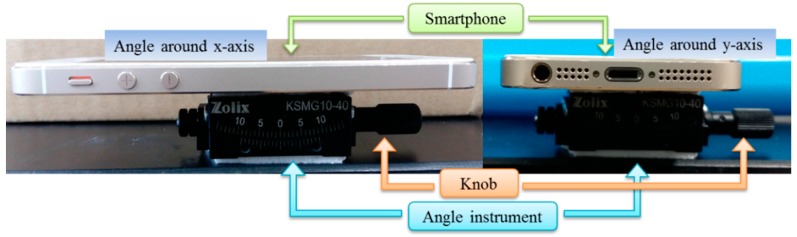
Photo of calibration experiment.

In order to validate the accuracy of the angle, an angle instrument was applied to test the angle variation. The experiment photo is shown as Figure 5.

**Figure 6:**
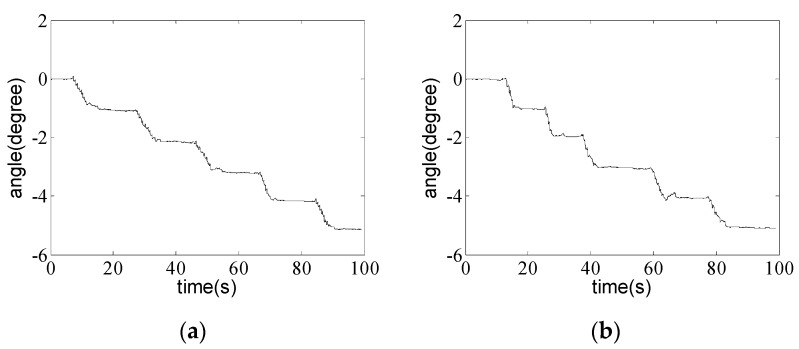
Angle variation with step-by-step method. (**a**) Angle around *x*-axis; (**b**) Angle around *y*-axis.

The angle instrument is fixed on the table, and the angle can be changed by turning the knob. The smartphone is fixed on the instrument, and moved with the instrument. Because of the dynamic angle data acquisition, slow manual angle loading, and low reading precise, step-by-step loading method is adopted in the experiment. Five loading steps are included in each test, one degree added every step by the angle instrument, and then keep static for several seconds, then goes to the next step. Take two experiments as examples, the angle around *x*-axis is shown in Figure 6a, and the angle around *y*-axis is shown in Figure 6b.

**Figure 7:**
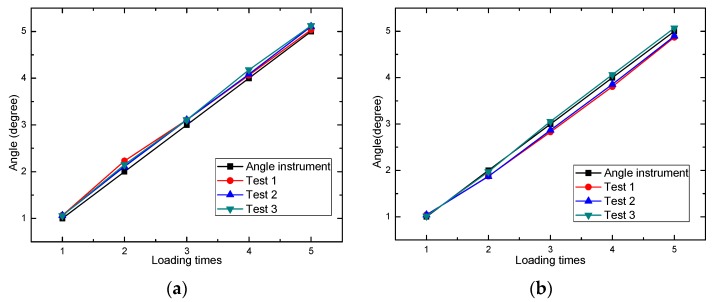
Angle step comparison between smart phone and angle instrument. (**a**) Angle step around *x*-axis; (**b**) Angle step around *y*-axis.

Several experiments were conducted, and angle steps on smart phone of three experiments are presented in Figure 7.

From Figure 7, it can be seen that the angle step coincides better with the instrument loading step. The error is inevitable because the angle on the instrument is loaded manually, and the reading is not so exact. Moreover, the loading in each experiment may be different to some extent, so the dynamic experiment was also conducted in [28]. The angle collected by the smartphone is compared to wired acquisition system and wireless sensing system, and the result proved the accuracy of angle acquisition using smartphone.

### 3.3. Controller Program

The controller program works with the web server to control and observe the data transmitted by the online collectors. The controller program can send instructions via the web server and make the collectors run based on these instructions. Three instructions are typically available: “start”, “stop”, and “upload data”. The “start” instruction automatically prepares the default settings and initiates the collection operation of online data collectors. The “stop” instruction controls online collectors, which are currently collecting data. The “upload data” instruction prompts online collectors to upload the data to the web server for further analysis.

Acting as the primary component of the system, the controller observes the online collectors, while the collectors collect data and returns six types of data (i.e., acceleration data of three directions and angle data of three directions) to the controller with their ID approximately every 20 s, it’s worth mentioning that the data will be returned to controller immediately once the collected data exceeds a given threshold. When the returned collected data exceeds a given threshold, the color of the corresponding monitoring parameter in the interface of the controller will change.

**Figure 8:**
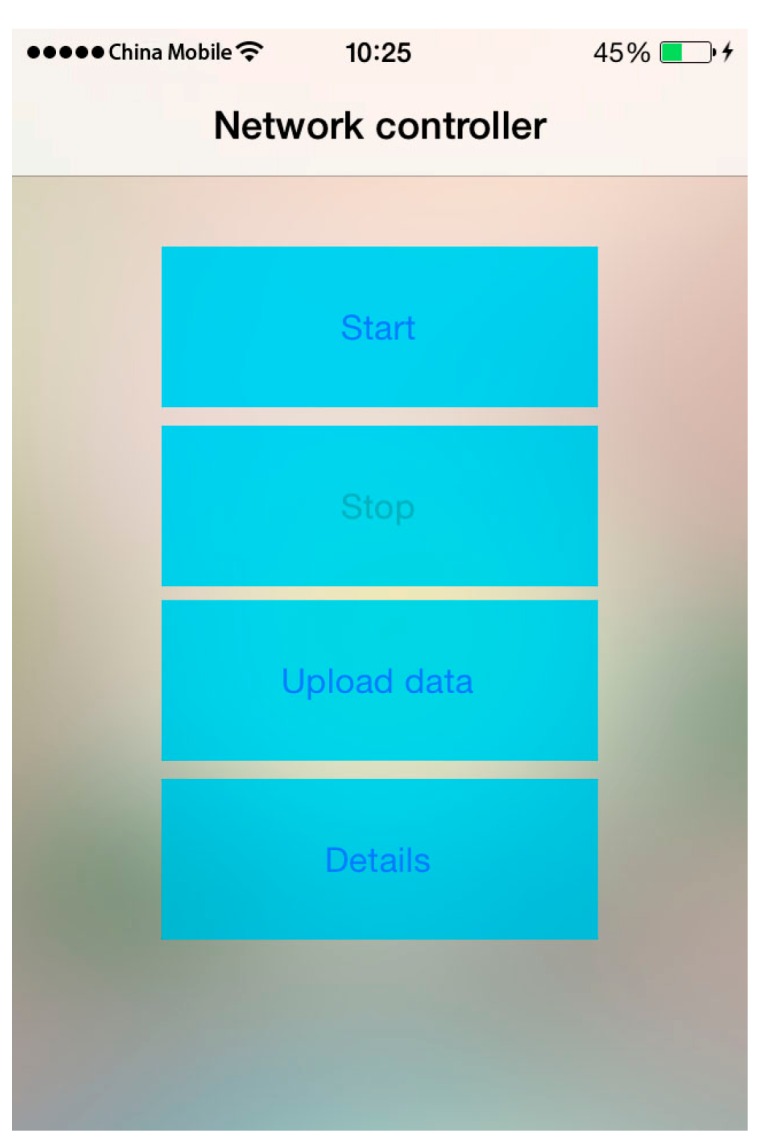
Controller interface.

**Figure 9:**
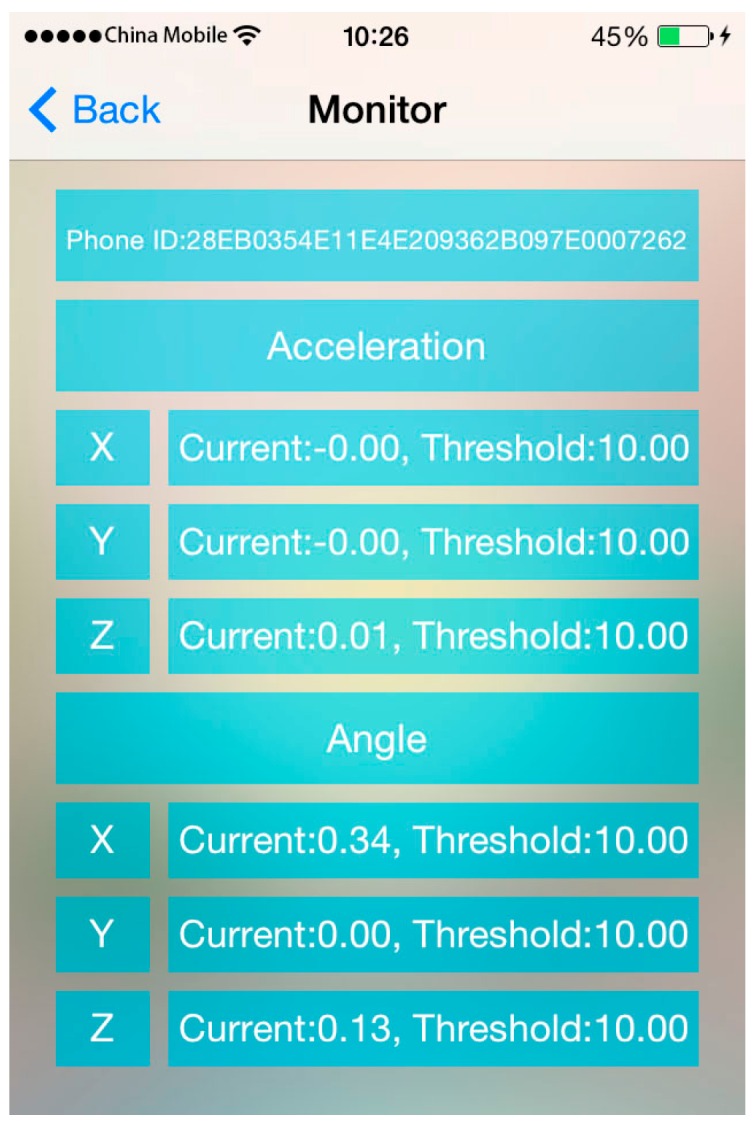
Interface of the real-time monitoring system.

The web server provides data storage and push functions, which facilitate information exchange (e.g., instructions) between the controller and the collectors. The interface of the controller is shown in Figure 8, and the interface of the real-time monitoring system is shown in Figure 9.

### 3.4. Collector Program

**Figure 10:**
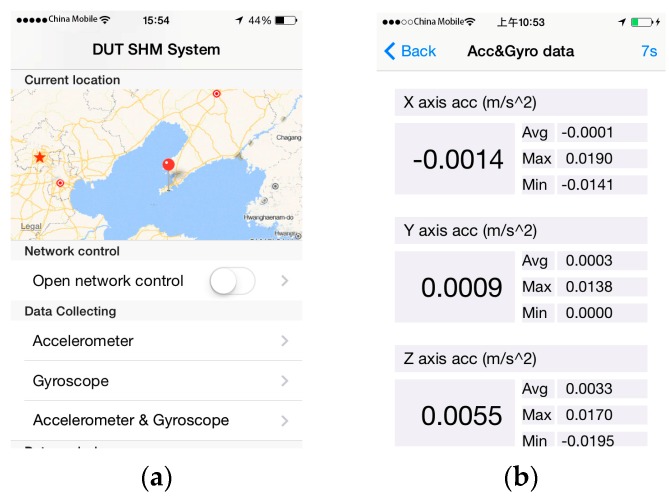
Collector interface of the iPhone. (**a**) GPS information; (**b**) Gyroscope interface.

The collector program uses the built-in sensors of the iPhone to collect angle and acceleration data. Prior to data collection, several variables can be set on a collector, including the collection frequency, duration, threshold, and data filename. During data collection, a collector continuously accesses its sensors to obtain data and writes their data to a file. A collector can work independently or under the control of the controller when “network control” is available. Additionally, a map position interface can be used to record the monitoring location. The interface of collector program is shown in Figure 10.

### 3.5. Application of the Proposed System for Monitoring a Hoisting Operation

**Figure 11:**
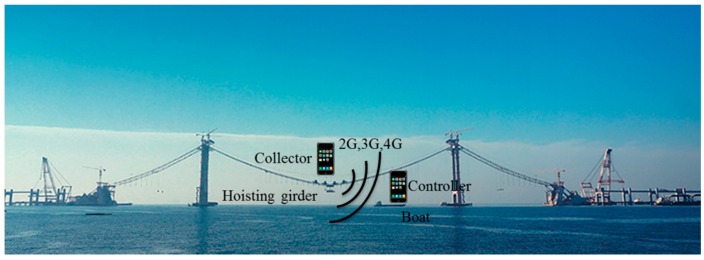
Schematic of the hoisting realization process.

The developed system was applied to determine the orientation of a girder during hoisting. A schematic of the hoisting process is shown in Figure 11. The collector was fixed on the girder to be hoisted, and the controller was positioned in a safe zone (i.e., a boat, on land, a catwalk, or another safe place), where operators can operate the system easily and safely. Instructions were sent from the controller to the collector via the 2G, 3G or 4G network. The collector then began to gather data upon receiving commands with a sampling frequency of 100 Hz and then returns six data at one time point (i.e., the acceleration data of three directions and the angle of three directions) to the controller every 20 s. The operator can determine the status of the hoisting procedure based on the returned data shown on the controller.

**Figure 12:**
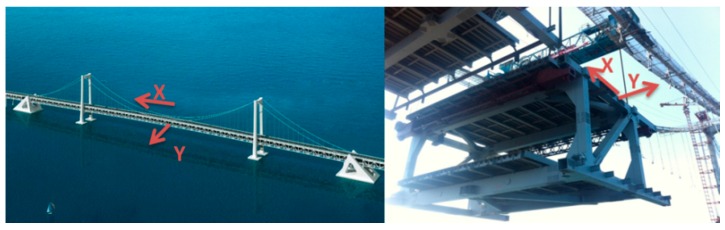
Specified directions.

The direction of the girder is specified as follows: the *x*-axis runs along the span of the bridge, and the *y*-axis is perpendicular to the bridge. These directions within the construction site are shown in Figure 12.

## 4. Monitoring of Side-Span Hoisting Procedure

### 4.1. Monitoring Process

**Figure 13:**
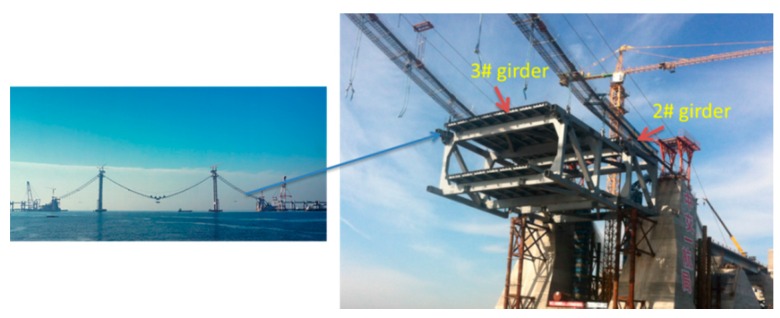
Location of the second girder.

The girder hoisting on one of the side spans was performed in this section. The hoisting tool used was a floating crane, and the girder, which is No. 3 in Figure 1, weighed 511.76 t. The location of the 3# girder is shown in Figure 13.

**Figure 14:**
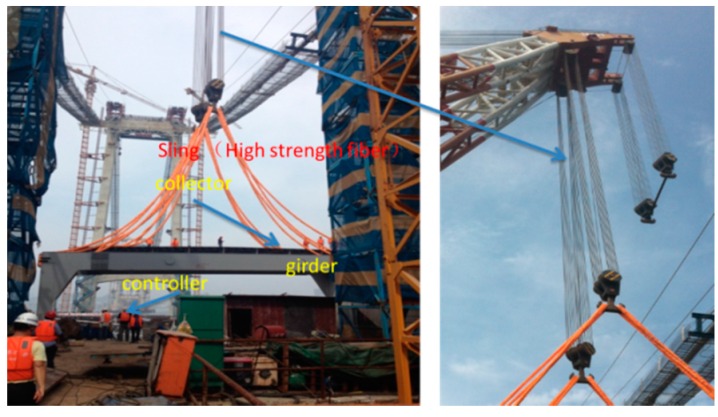
On-site hoisting.

**Figure 15:**
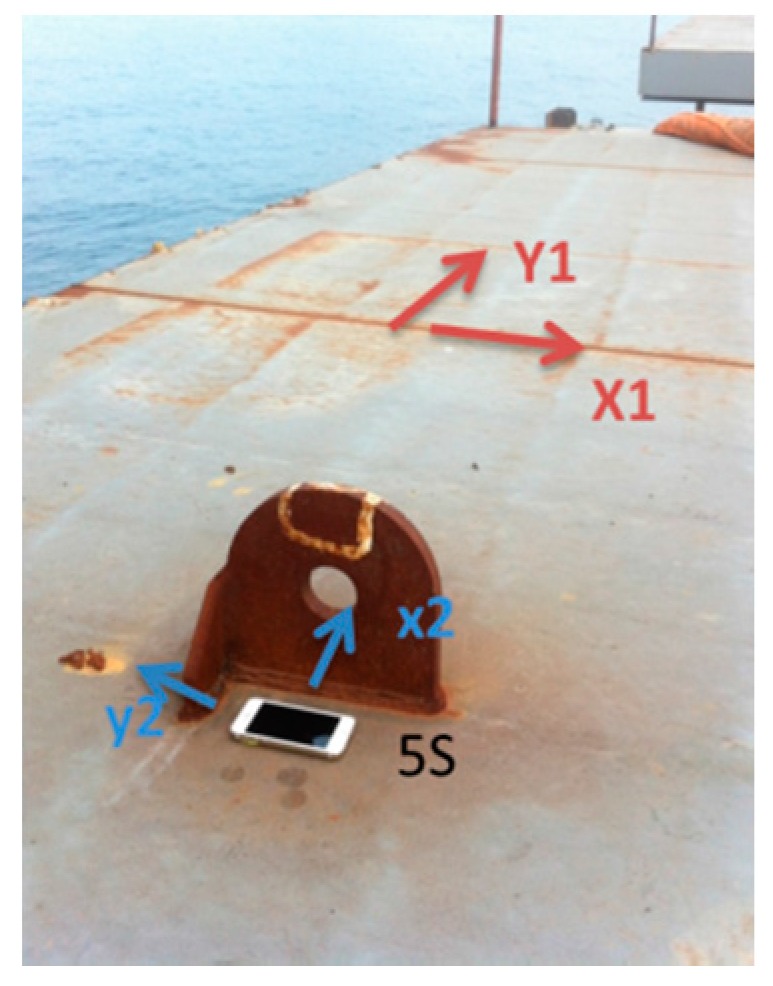
Arrangement of the collector (i.e., the iPhone 5S).

During hoisting the girder is lifted by slings that are made of a high-strength fiber. The orientation of the girder when it is suspended requires monitoring to prevent the sudden drop of one end, which would be indicated by a large angle in the front/back or right/left directions. Therefore, to obtain this real-time information and then take corresponding measures, the angle and acceleration should be monitored and relayed to the operator through the controller. During the hoisting process, an iPhone 5S gathered data as a collector, while an iPhone 4S sent instructions and received data as a controller. The on-site hoisting procedure is shown in Figure 14. The collector (i.e., the iPhone 5S) was fixed on the girder, and the controller (i.e., the iPhone 4S) was operated by a worker on a safe place. The arrangement of the phone is shown in Figure 15. The directions X1 and Y1 denote the positive directions of the *x*- and *y*-axes of the girder, and the directions X2 and Y2 represent the positive directions of the *x*- and *y*-axes of the collector. Thus, the returned data on the angle about the axis of the girder should be considered during hoisting.

### 4.2. Related Algorithm

**Figure 16:**
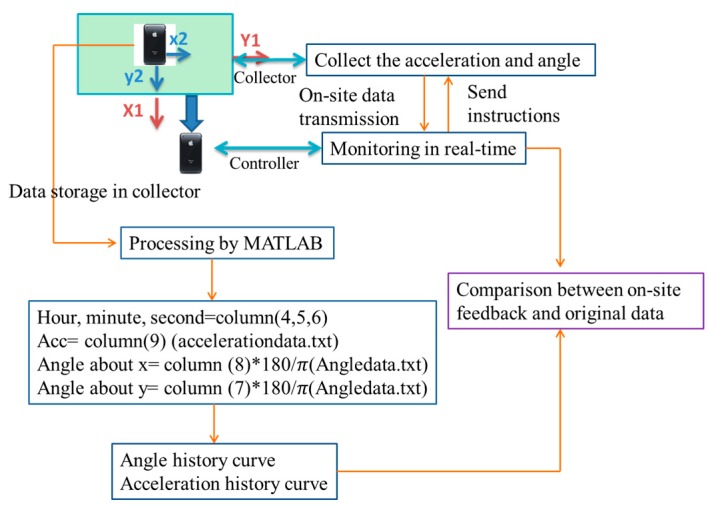
Algorithm of side-span hoisting.

In order to compare the on-site feedback and original data collected by collector, the related algorithm is shown in Figure 16. The data is collected and stored in the collector phone, and named as Angledata.txt and acceleration.txt. The data files can be exported, and then processed by MATLAB to obtain the time-history curves. In the data file, the first to the sixth column represents collected time, they are year, month, day, hour, minute and second respectively. In this test, hour, minute and second are used. In the accelerationdata.txt file, the seventh to ninth columns represent the acceleration of three axes, so the vertical acceleration is the ninth columns. In the Angledata.txt file, the seventh to ninth columns represent the angle around three axes. From Figure 15, it can be seen that, the angle around *x*-axis of the girder measured by smartphone is the angle around *y*-axis. So the eighth and seventh column is used to calculate the angle around *x*-axis and *y*-axis respectively. The original data in smartphone collected in radians, a transformational relation is used in the data processing. After the processing, the angle-history curve and acceleration history curve can be obtained to compare with the on-site feedback.

### 4.3. Test Results

**Figure 17:**
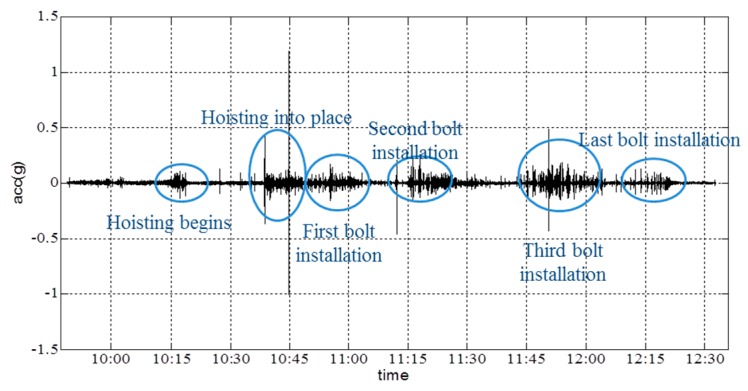
Vertical-acceleration time-history curve of the girder during hoisting.

The duration of the hoisting process was 3 h. There was no warning information on the controller during the whole procedure. At the beginning, the four corners of the girder must be hoisted simultaneously. Therefore, real-time angle information must be obtained to evaluate the position of the girder and to provide information to operators. The vertical-acceleration time-history curve of the collector is shown in Figure 17.

**Figure 18:**
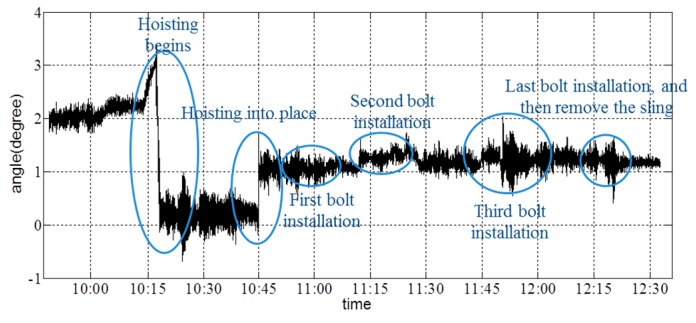
Angle about the *x*-axis of the girder during hoisting.

**Figure 19:**
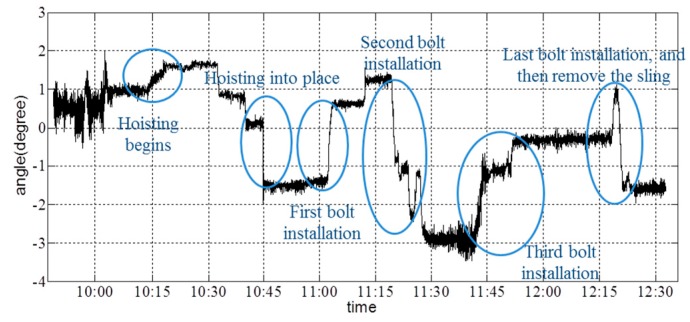
Angle about the *y-*axis of the girder during hoisting.

From the vertical-acceleration time-history curves in Figure 17, it is shown that the acceleration fluctuated to a certain extent when the girder state changed. Additionally, the maximum acceleration occurred when the girder is hoisted into its final location; the sudden touch of the girder and the column caused a major acceleration. Figure 18 and Figure 19 show the angle about the *x*- and *y*-axes of the girder.

**Figure 20:**
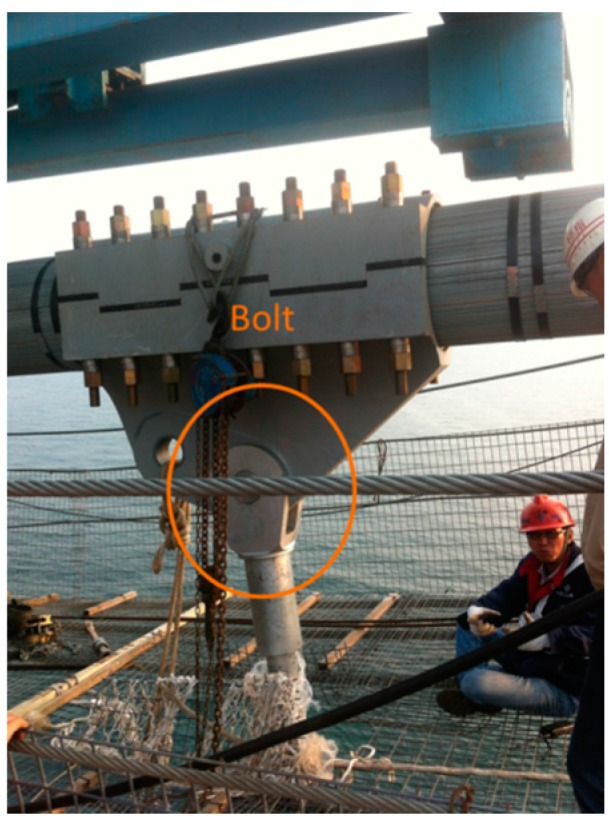
Bolt.

Depending on the records of the field operator, the collector started to gather data at 9:48; the hoisting of the girder began at 10:14; the girder was lifted to a given height by 10:45; and four bolts (i.e., the connection between the cable and girder, which is shown as Figure 20) were installed completely by 12:19. The resultant deformation was severe when workers started to remove the sling. From Figure 17, Figure 18 and Figure 19, it is shown that the angle changed due to the action of hoisting in every phase of the hoisting procedure. Before each bolt was secured in its respective hole, the angle was adjusted to match up each bolt hole. Throughout the hoisting process, the acceleration and angle changed only marginally, and all disturbances remained within the acceptable ranges. Based on the preceding analysis, the girder remained stable during this hoisting operation, which is consistent with the phone investigation.

## 5. Monitoring of a Middle-Span Hoisting Procedure

### 5.1. Monitoring Process

The girder which is No. 22 in Figure 1 on the middle span weighed 372.22 t and was hoisted using two cranes. Two cranes were stretched across the two primary cables and worked together to lift the second girder into place; thus, both cranes had to raise the girder at the same rate to prevent imbalance. Therefore, the angle of the girder must be determined in real time to ensure a synchronous hoisting operation.

**Figure 21:**
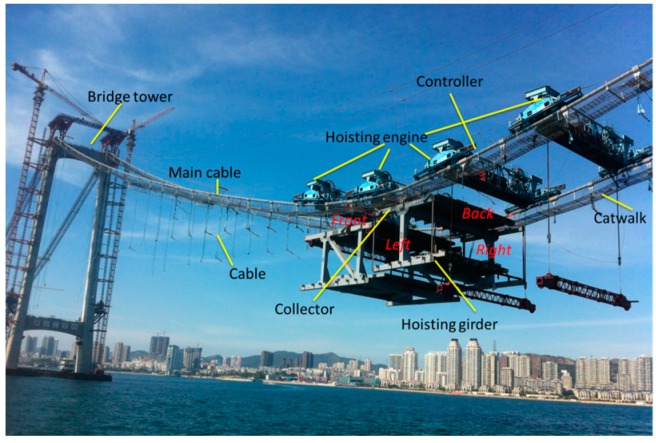
On-site hoisting of the middle span.

**Figure 22:**
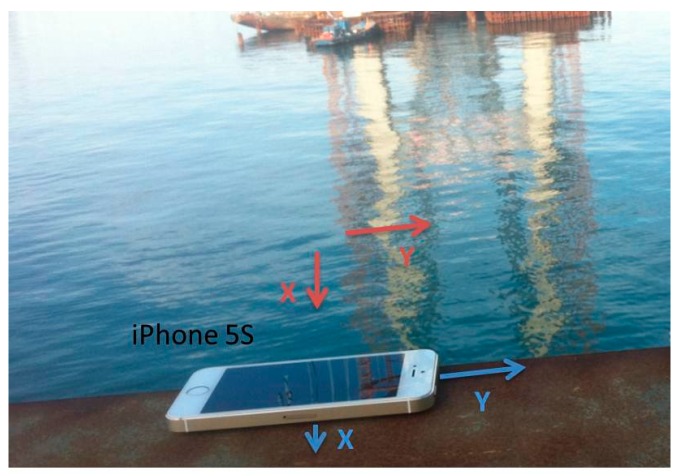
Arrangement of the collector.

**Figure 23:**
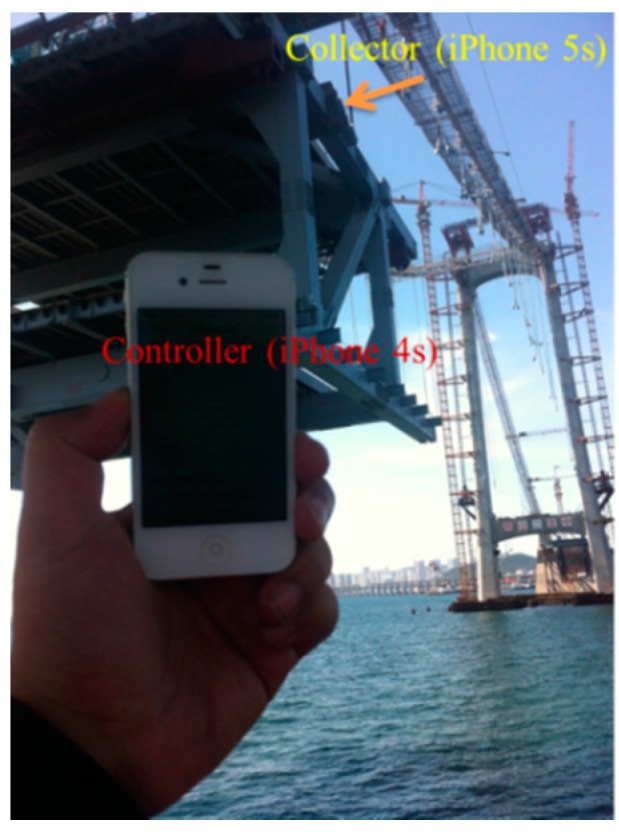
Position of collector and controller.

In this monitoring procedure, an iPhone 5S was used to collect data and return information to the controller, and an iPhone 4S served as a controller to receive the returned data and provide information to the operators. The on-site hoisting procedure is shown in Figure 21. The collector (i.e., the iPhone 5S) was fixed onto the girder to be hoisted, and the controller was operated by operators on a catwalk. The arrangement of the collector is shown in Figure 22, and Figure 23 shows the position of the collector and controller at the beginning of the hoisting operation; at this point during the hoisting procedure, the controller was on the boat. After the girder was raised from the boat, the controller was taken to a catwalk for the remainder of the hoisting procedure.

As shown in Figure 22, the collector was pointed toward a direction similar to the specified direction of the truss girder to allow operators to judge the monitored situation directly based on the feedback information received by the controller. Figure 23 shows the convenience of this method, without any other measurement devices; only two smart phones are required to obtain relevant hoisting information.

### 5.2. Related Algorithm

**Figure 24:**
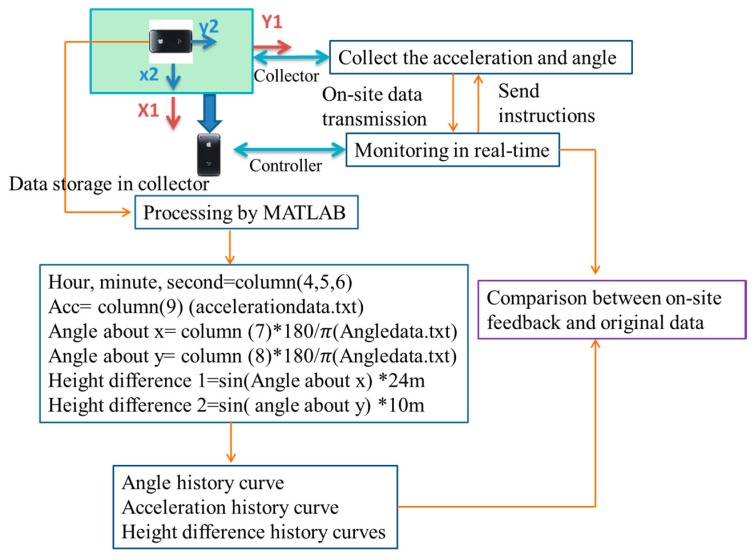
Algorithm of middle-span hoisting monitoring.

Figure 24 gives the algorithm of the middle-span hoisting monitoring. The detailed accounts are omitted because of which are similar to the algorithm in Section 4.2 except for two aspects. First is the same direction of girder and smartphone, so the seventh column is used to calculate the angle about *x*-axis, and the eighth column is used to calculate the angle about *y*-axis. Second is regarding the size and angle of the girder, the height difference in the front/back and right/left directions can only be obtained approximately. The height difference in the left/right direction can provide some information to the hoisting operators about the synchronization of the cranes on the two primary cables. The girder was 24 m long in the left/right direction and 10 m wide in the front/back direction, so the height difference can be calculated according to trigonometric function shown as Figure 24.

### 5.3. Test Results

The duration of the entire hoisting process was nearly 9 h—from the transmission of the “start” instruction at 9:00 to the transmission of the “stop” instruction at 17:52. The test period is so long and the battery in iPhone can function 4 h in working state, while this long field test can be achieved if an additional battery is available.

**Figure 25:**
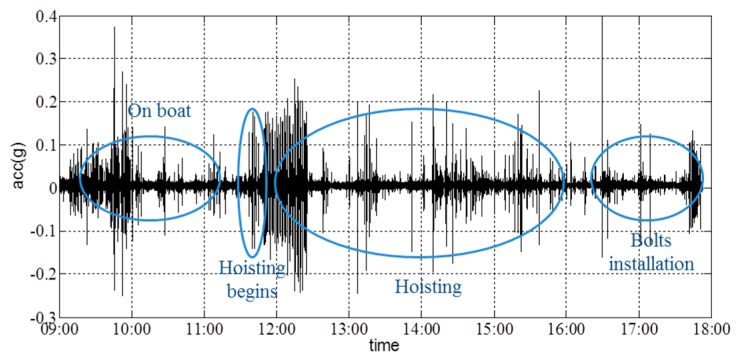
Vertical-acceleration time-history curve.

The vertical acceleration collected by the collector is shown in Figure 25 and shows that there is no significant change in the vertical acceleration of the girder. The maximum acceleration occurred at the beginning of the hoisting operation.

**Figure 26:**
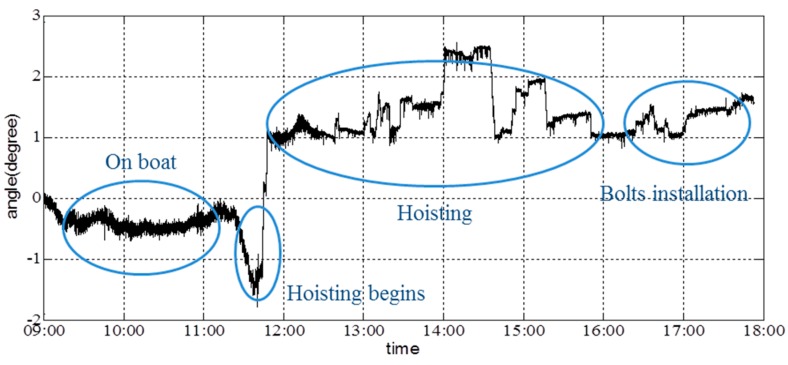
Angle about the *x*-axis of the girder.

**Figure 27:**
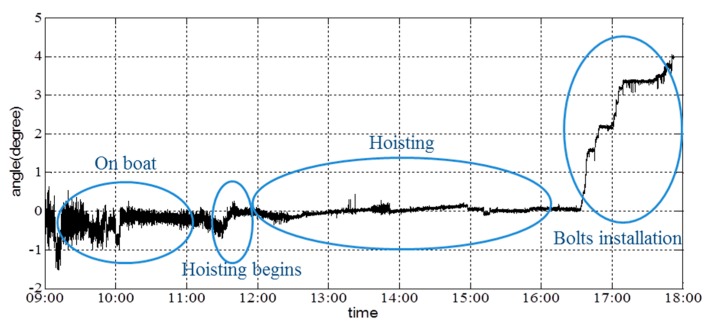
Angle about the *y*-axis of the girder.

Figure 26 shows the angle about the girder’s *x*-axis; the angle about the *y*-axis of the girder is shown in Figure 27. Data collection began at 9:00, and the hoisting process began at 11:31. A certain angle about the *x*- and *y*-axes was generated at this time. During the initial phase of hoisting, the angle changed marginally to ensure synchronous hoisting of the two sides. Additionally, the first bolt was placed in position at 16:30. The angle about the *y*-axis continued to change to match the installation of the other three bolts. Based on the figures, it is shown that the acceleration and angles can be reported in real time. Lack of synchronization was not encountered during this hoisting operation.

**Figure 28:**
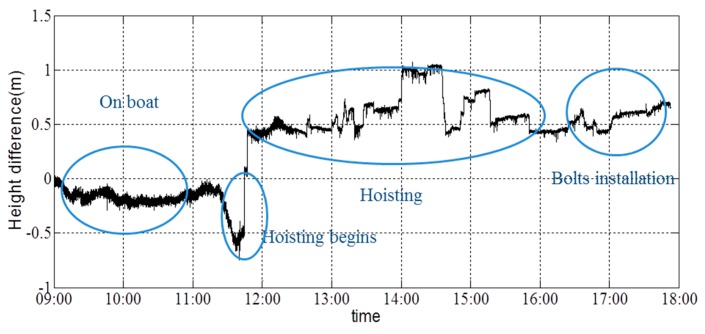
Height difference in the left/right direction.

**Figure 29:**
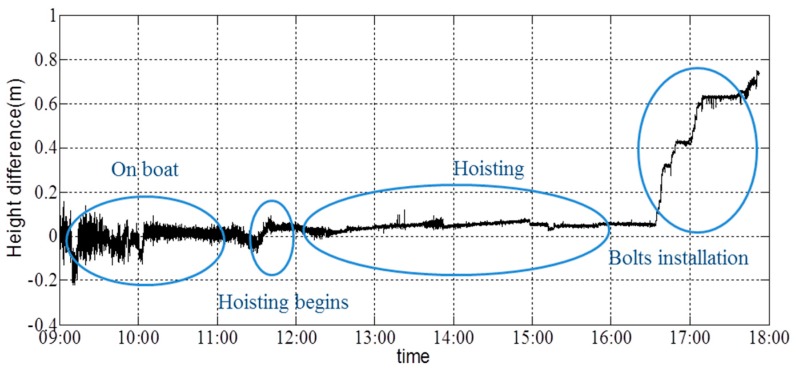
Height difference in the front/back direction.

The height difference recorded in the left/right direction is shown in Figure 28, and the height difference recorded in the front/back direction is shown in Figure 29. It is shown that the height difference in the left/right direction was adjusted to ensure the synchronization of the two cranes. At the end of hoisting process, the height on the left side was 0.7 m higher than that on the right side. The height in the front/back direction was approximately stable throughout the hoisting operation. After the first bolt was installed, the height in the front/back direction continued to increase to allow the installation of the three other bolts. After the installation of all of the pins, the height in the front of the girder was 0.75 m higher than that in the back of the girder. No alarm information appears during the on-site monitoring. Thus, the hoisting process was successful, and the last state of the girder had been prepared completely for the construction of the next day, and there is no warning information on the controller during the whole procedure.

## 6. Conclusions

In this study, a CPS iPhone-based system for hoisting monitoring using smartphones was proposed, including a phone collector, a controller and a server. Field monitoring results of two steel girder elements of a cross-sea bridge confirmed the convenience and practicability of this system. The dynamic acceleration and angle data of both hoisting procedures were collected and sent to a web server. The functions of sending instructions to the collectors and real-time monitoring can significantly enhance field hoisting monitoring capabilities. The changes in various angles and height differences obtained during the hoisting procedures were all within normal ranges, and there is no warning information, which indicates that the girder hoisting process was stable and safe. The real-time monitoring feedback received by the control system can provide important information to operators so that adjustments can be made during hoisting if necessary. The use of smartphone has advantages, including convenience, lower cost, the use of commonly available smartphones, and intuitive operation. Additionally, the proposed monitoring system is simple enough to be operated by any construction worker equipped with an iPhone. The rapid popularity of smart phones has provided the opportunity for researchers and civilians to understand traditional monitoring methods in a new way. For the aforementioned advantages, the use of smartphones will hopefully represent a promising trend in the CPS field.

## Figures and Tables

**Table 1:** Basic features of the accelerometer and gyroscope in the iPhone.

	Accelerometer (BMA220)	Gyroscope (L3G4200D)
Supply voltage	1.62–1.98V	2.4–3.6 V
Low voltage-compatible IOS	1.8 V	1.8 V
Data output	16 bit	16 bit
Selectable full scales	±2 g/±4 g/±8 g, ±16 g	250 dps/500 dps/2000 dps
Output interface	I^2^C/SPI	I^2^C/SPI
High shock survivability	Yes	Yes

**Table 2:** Mechanical characteristics of the accelerometer in the iPhone 5S.

Parameter	Conditions	Typical
Measurement range (MR)		±2, ±4, ±8 g, ±16 g
Sensitivity	±2.0 g	16 LSB/g
	±4.0 g	8 LSB/g
	±8.0 g	4 LSB/g
	±16.0 g	2 LSB/g
Sensitivity change vs. temperature	±2.0 g	±0.01%/°C
Typical zero-g offset accuracy	±2.0 g	±95 mg
Operating temperature range		−40 to +85 °C
Zero-g offset temperature drift	−40 to +85 °C	±2 mg/K
Bandwidths		32, 64, 125, 250, 500, 1000 Hz

**Table 3:** Mechanical characteristics of the gyroscope in the iPhone 5S.

Parameter	Test Conditions	Type	Unit
MR		±250, ±500, ±2000	dps
Sensitivity	MR is ±250 dps	8.75	mdps/digit
	MR is ±500 dps	17.50	
	MR is ±2000 dps	70	
Sensitivity change vs. temperature	−40 °C to +85 °C	±2	%
Digital zero-rate level	MR is ±250 dps	±10	dps
	MR is ±500 dps	±15	
	MR is ±2000 dps	±75	
Zero-rate level change vs. temperature	MR is ±250 dps	±0.03	dps/°C
	MR is ±2000 dps	±0.04	
Digital output data rate		100, 200, 400, 800	Hz
